# Preparatory planning framework for Created Out of Mind: Shaping perceptions of dementia through art and science

**DOI:** 10.12688/wellcomeopenres.12773.1

**Published:** 2017-11-06

**Authors:** Emilie Brotherhood, Philip Ball, Paul M Camic, Caroline Evans, Nick Fox, Charlie Murphy, Fergus Walsh, Julian West, Gill Windle, Sarah Billiald, Nicholas Firth, Emma Harding, Charles Harrison, Catherine Holloway, Susanna Howard, Roberta McKee-Jackson, Esther Jones, Janette Junghaus, Harriet Martin, Kailey Nolan, Bridie Rollins, Lillian Shapiro, Lionel Shapiro, Jane Twigg, Janneke van Leeuwen, Jill Walton, Jason Warren, Selina Wray, Keir Yong, Hannah Zeilig, Sebastian Crutch

**Affiliations:** 1 Dementia Research Centre, Institute of Neurology, University College London, London, UK; 2 Created Out of Mind, The Hub, Wellcome Collection, London, UK; 3 Science Writer, London, UK; 4 Salomons Centre for Applied Psychology, Canterbury Christ Church University, Tunbridge Wells, Kent, UK; 5 Tryce Ltd., London, UK; 6 Visual Artist, London, UK; 7 British Broadcasting Corporation, Portland Place, London, UK; 8 Royal Academy of Music, Marylebone, London, UK; 9 Dementia Services Development Centre (DSDC), DSDC Wales, Bangor University, Bangor, UK; 10 Collaborate, Clarence Centre for Enterprise & Innovation, St George’s Circus, London, UK; 11 Department of Computer Science, Faculty of Engineering Science, University College London, London, UK; 12 Living Words, The Workshop, Folkestone, Kent, UK; 13 Rare Dementia Support, London, UK; 14 National Youth Choirs Great Britain, The Rivergreen Centre, Durham, UK; 15 Wellcome, Euston Road, London, UK; 16 Department of Molecular Neuroscience, University College London, London, UK; 17 London College of Fashion, University of the Arts London, Marylebone, London, UK

**Keywords:** Dementias, creative arts, interdisciplinarity, methodology, disciplines

## Abstract

Created Out of Mind is an interdisciplinary project, comprised of individuals from arts, social sciences, music, biomedical sciences, humanities and operational disciplines. Collaboratively we are working to shape perceptions of dementias through the arts and sciences, from a position within the Wellcome Collection. The Collection is a public building, above objects and archives, with a porous relationship between research, museum artefacts, and the public.  This pre-planning framework will act as an introduction to Created Out of Mind. The framework explains the rationale and aims of the project, outlines our focus for the project, and explores a number of challenges we have encountered by virtue of working in this way.

## Background and rationale

### The Hub Award

The Created Out of Mind project constitutes the second interdisciplinary group taking up residency at The Hub at Wellcome Collection. The Hub affords the opportunity to stretch the definition of what collaboration means, with the award encouraging the bringing together of professionals from varying backgrounds, including academia, the creative arts and communication. Residents work on a two year project aiming to: explore health in its cultural and social contexts, develop a programme of public engagement, create work that is original and impactful, and produce new insights, forms of engagement, methods for conducting research or interventions. From October 2014 to October 2016, the first Hub residents ‘Hubbub’ (led by Durham University) explored the dynamics of rest, activity and work, as they operate in mental health, the neurosciences, the arts and the everyday ( Callard *et al.*, 2016).

### The Created Out of Mind residency at The Hub (2016–2018)

Created Out of Mind is a team aiming to explore, challenge and shape perceptions and understanding of dementias through science and the creative arts. The team will explore what dementia means to us all, challenge traditional definitions and common misconceptions, and unlock what we can learn about art, consciousness and the brain from the experiences of people with dementia. Created Out of Mind is led by a core team consisting of project director Sebastian Crutch (neuropsychology), and co-directors Caroline Evans (management, strategy), Philip Ball (science writing, broadcasting), Paul Camic (psychology, public health), Nick Fox (neurology, neuroimaging), Charlie Murphy (visual arts), Fergus Walsh (journalism), Julian West (music practice, education), and Gill Windle (gerontology).

### The context of conducting an interdisciplinary project

Collaborative working shines a light on the detail, the operational, the mundane and those seemingly everyday encounters that take place during a project such as Created Out of Mind, where individuals and practices rub against each other. The Hub brings the politics of the arts, humanities, sciences and professional practice to the fore. It holds these disciplines and roles in a troubling middle-ground, whereby the discomfort of individuals often produces new and divergent approaches to the questions being asked.

Today, interdisciplinary work is widely advocated, generally considered to be a “good thing”, but often conducted without any real consensus about what it should mean. To some, this cross-pollination is a critical element in the transferral and expansion of the collaborator’s practices and the production of innovative knowledge, public discourse and research; others argue that a new mindset and perhaps even new institutional, academic and intellectual structures are needed to tackle the many important problems that fall outside of, or span, conventional disciplinary boundaries. Is interdisciplinarity something expressed in the outlooks of individuals, or via the collaboration of specialized teams?

While there may be no one-size-fits-all resolution to such questions, interdisciplinary work, if conducted well, has the potential not just to create new avenues for tackling existing problems that cannot be addressed by a single discipline alone, but also to identify entirely new questions, problems and methodologies.

### Rationale for the focus of Created Out of Mind

Dementia is perhaps best understood as a catch-all phrase that refers to several hundred different conditions that are characterised by progressive cognitive decline which impacts on behaviour, affect and motivation ( WHO, 2012). It is frequently presented as one of the leading health and socio-economic challenges of our age ( WHO, 2012), with a massive increase in new cases predicted (more than 35 million people worldwide, a number that is expected to almost double every 20 years; G8 Dementia Summit Declaration, 2013). Nevertheless, the numbers of people living with a dementia are beginning to effect a cultural transformation. People living with a dementia are increasingly uniting to advocate for themselves and receiving greater public awareness. The prevalence of the condition is influencing everyone from law makers (with dementia considered a human rights issue, in regard to the public discourse on assisted dying), town planners (in terms of work on the citizenship of people with a dementia ( Kontos *et al.*, 2017 and Shakespeare *et al.*, 2017) and systemic changes regarding dementia-friendly communities) to theatre directors (“Dementia has now become what daffodils were to Wordsworth to some of the keenest artistic minds of our era”, Paul Taylor review of Plaques and Tangles in The Independent, 21 October 2015 ( Taylor, 2015)). Aspirations and fears are also shifting, with the focus of many people moving from living longer to living well, and concerns about dementia exceeding that of cancer, heart attack or stroke ( YouGov survey, 2011, commissioned by Alzheimer’s Society and Saga Healthcare). We now urgently need new insights, new means of exchange, new approaches to working and methods of evaluation, and new engagement between scientific, cultural and political leaders not just to improve public engagement but to equip all of society to cope with this changing cultural landscape.

**Figure 1.**
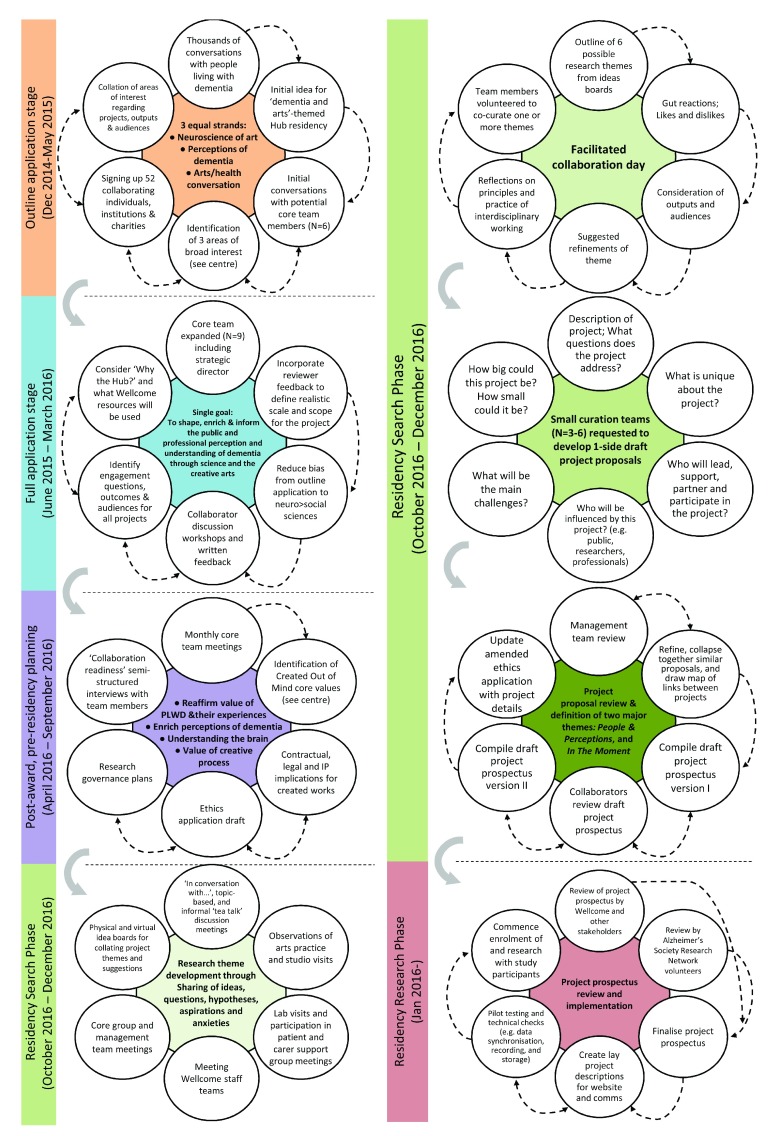
Summary timeline of actions and developing topics throughout the application, planning, search and initial research phases of the Created Out of Mind residency.

The creative arts are a powerful vehicle for understanding, communicating and navigating this dramatic cultural shift. This potential is apparent in individuals who have had their creative abilities/interests blunted by dementia: the artist who loses the ability to perceive form and space, and the writer who can’t find her words (For further reading, please see Crutch *et al.*, 2001; Garrard *et al.*, 2005). Many people with deteriorating cognition pick up new artistic media to explore and convey their experiences, with some arguably even released into fresh creativity as a direct consequence of their degenerative illness ( Byers, 2011; Kapur *et al.*, 2013; Young *et al.*, 2016; Zeilig *et al.*, 2014; see Figure 1). Artistic change in neurodegenerative conditions also provides powerful insights into the brain mechanisms underpinning specific art forms such as musical cognition ( Fletcher *et al.*, 2015), as well as more general cultural attributes such as creativity and aesthetics ( Halpern *et al.*, 2008; Halpern & O’Connor, 2013).

Recently, with increased recognition of the health, well-being and socioeconomic impacts of dementia, there has been a proliferation of cultural initiatives for engaging, stimulating and educating people with dementia and their carers ( Zeilig *et al.*, 2015). The All Party Parliamentary Group on Arts, Health and Wellbeing’s newly released inquiry report highlighted the impact and importance of arts participation in dementia care, and has recommended cross-sector policy, research and practice initiatives ( APPG, 2017). In addition, critical reviews of the arts for dementia care suggest that participatory art interventions have the potential to enrich quality of life and communication ( de Medeiros & Basting, 2014; Mental Health Foundation, 2011; Salisbury *et al.*, 2011; Young *et al.*, 2016; Zeilig *et al.*, 2014). Nearly all art forms offer people with dementia and carers opportunities for creative expression, skill development, cognitive and emotional stimulation, and non-stigmatising social engagement. In particular, research involving singing ( Engström *et al.*, 2011; Särkämö *et al.*, 2013), music ( Ragneskog *et al.*, 2001; van der Vleuten, 2012), visual arts ( Camic *et al.*, 2016; Windle *et al.*, 2014) and museum programmes ( Camic *et al.*, 2017; Johnson *et al.*, 2017) have shown promising initial findings.

In terms of the role of the arts in dementia care, despite the existence of considerable practice innovations, the critical research reviews identify that in many instances the benefits are often insufficient and tentative, especially for the visual arts. This is attributed to design limitations in some studies, but also because ‘the field is still in its infancy’ ( Zeilig *et al.*, 2014) and requires further research development. This speaks to the fact that the scientific study of dementia and artistic explorations into its effects rarely overlap. Dementia and arts practice and research seldom reflect the fact that different dementias target different brain regions and yield different symptoms from visual problems to personality change to language difficulties. Consequently little is known about how, why or whether such activities affect the lives of those concerned. How do different forms of dementia influence artistic expression and appreciation? Which artistic form is most appropriate for whom? How does engaging with the arts facilitate the creativity of people living with a dementia? How long do benefits last? How might the arts be used as communicative tools in dementia care? Do we need new approaches and methods to evaluate such cultural activities? Do the arts have a valid role in dementia care when only limited medical treatments are available? If so, should health and social services pay for them? And what burden of proof should they require?

## Study conception and design

### *Why a ‘*Preparatory planning framework’ *? Search before research*

*‘Science is hypothesis driven…But the first step on this journey, namely the generation of the hypothesis itself, is rarely discussed…. As physicist … Erwin Schrodinger put it, “The task is not to see what has never been seen before, but to think what has never been thought before about what you see every day”. This early and ill-described stage entails a ‘methodology gap’. It is at this messy and amorphous juncture, where the creation of knowledge starts, that scientists must find their inspiration. It is an opening that offers opportunities to collaborations between art and science*.

*Artists’ productivity, like scientists’, comes from an attitude of curiosity, an urge to find out. However, in artistic endeavour, it is the methodology gaps, rather than the methodology itself, that are often most evident.’* (From Bergit Arends’ and Davina Thackara’s Preface to *Experiment: Conversations in art and science*, p10-11 ( Arends & Thackara, 2003))

A critical component of The Hub proposition is to engage in a genuinely interdisciplinary programme of research. Indeed, one senior figure involved in the residency selection process suggested that to deliver precisely on the plans outlined in the Hub award application would be disappointing, such was the appetite for something genuinely exploratory, collaborative and creative. Working in this way requires building familiarity, understanding and trust between collaborators with different backgrounds, languages, interests, motivations and ways of working. It also requires that what Arends and Thackara refer to as ‘generation of the hypothesis’ is a collaborative, joint activity, not one undertaken by a subset of individuals within the partnership ( Arends & Thackara, 2003). Consequently the initial set of ideas for the research – or protocol - for the Created Out of Mind project was written during the first 3 months of the residency. Commencing the residency without a fixed plan required holding the tension between the common scientific practice of executing a pre-determined set of aims, procedures and analyses (research), and an approach arguably more common in artistic practice based on discovery through exploration in which the process may be as important as the end product (search). The protocol was developed through the following stages (see summary of timeline in Figure 1):

- Project was conceived by the grant holders (SC, PB, PC, CE, NF, CM, FW, JW, GW) and elaborated with the other authors.
- Ideas, questions, themes, hypotheses, written and drawn contributions, and aspirations were collated and shared via means of:
- Initial conversations, collaborator interviews, observations of arts practice, visits to scientific laboratories, support group meetings with people living with different forms of dementia and their carers, presentations to Wellcome staff teams, core group meetings and wider collaborator discussion meetings. These activities were then distilled into:
- Six themed project boards concerning the topics of current arts practice, cultural spaces, aesthetics, language, available evaluation tools and public engagement. These formed the basis for a subsequent:
- Facilitated collaborator event involving the authors and comprising guided discussion of the six themed areas (gut reaction, likes, dislike, refinements, outputs, actions) and broader reflections on topics such as interdisciplinary working and the role of people with dementia across the themes. At the end of this meeting:
- Small groups of 4–6 collaborators curated each themed area.
- Curation teams subsequently and collectively wrote descriptions of individual proposed projects using a template form.
- Project proposals were initially vetted by members of the Created Out of Mind management team (SC, CE, EB) who reviewed the projects by suggesting combinations of overlapping proposals, drawing connections between projects, and identifying work requiring further development or external collaboration. This process collapsed the six initial themes down to two: *People and Perceptions*, and *In the Moment*.
- A collated prospectus of proposals was then circulated to all authors for amendment, clarification and approval.
- Further feedback and improvements were then re-sought from Wellcome staff and partner individuals and organisations.

## Emerging principles from the ‘search’ phase which influenced ‘research’ design

### Experiment vs Experience

A central aim of the Created Out of Mind project is to develop novel toolkits, ways of conducting research, new questions and ways of thinking which enable us to better understand the role of and ways of using the arts with people living with dementia. To reflect the diversity of arts and cultural practice and the number of different scenarios in which evaluation may be of value, three broad areas of arts-based activity will be explored in the current study: (i) individual responses to artistic stimuli in an experimental setting (e.g. eye-tracking based studies of viewing visual art, object handling, listening to music), (ii) individual and group responses to participatory and co-creative arts activities (e.g. choir, improvisatory music making, drawing or painting objects); and (iii) unguided individual exploration of cultural spaces (e.g. viewing galleries or museum collections).

By introducing elements of evaluation and monitoring into arts-based activities, one risk is that the situation is fundamentally altered. Responses may differ considerably depending on whether participants feel like they are the subject of a controlled arts-based experiment or a recipient of and/or contributor to an authentic arts experience. In order to navigate this experiment-experience continuum, the visibility and extent of evaluation must be varied within the different arts-based activity settings. This will be achieved through (a) use of discreet monitoring instruments which minimise interference with the activity (e.g. simple wearable technologies such as wristbands or sensors fixed to clothing), (b) the inclusion of control trials conducted in the absence of any overt monitoring or measurement, and (c) the recording of arts activity sessions using an unobtrusive 360-degree video camera.

Responses to arts-based activities will also be influenced not only by the physical environment but also the social environment. For example, systematic reviews of a wide range of arts- and non-arts-based psychosocial interventions have underlined the value formal carers place upon interventions focusing on getting to know, understand and connect with residents with dementia ( Rapaport *et al.*, 2017). Evaluations of arts-based practices such as singing in dementia have shown a primary impact of some experiences may be upon the quality of relationship between the person with dementia and members of their support network who have also participated in the activity (e.g. sense of togetherness; Unadkat *et al.*, 2017). Social influences upon responses will be examined not only through the use of both individual and group activities, but also by the inclusion of social conditions within individual response experiments (e.g. viewing visual art in isolation vs with facilitation vs with recordings of others’ responses to the same artefact).

### Complementary qualitative and quantitative evaluation

Attempting to capture, describe or communicate something of the experience of people with different forms of dementia as they participate in arts activities naturally led to early debate amongst collaborators as to whether our work is better suited to quantitative or qualitative methodologies. As recently noted, ‘Quantitative experimental research can investigate whether an arts intervention is capable of producing a desired change in the health status of participants. However qualitative research is not well-suited to measuring effects. Hence qualitative researchers do not tend to talk about outcomes or variables, but rather phenomena in specific contexts. Qualitative research is useful for exploring experience and perspectives and to explore novel connections and under researched phenomena ( Daykin & Stickley, 2015, p73).

A host of quantitative tools exist for studying human behaviour in complex social environments. Many of these tools are designed specifically to understanding the role and contribution of the arts to the lives of people living with dementia and their carers in health, social care, cultural and community settings (e.g. visual analogue rating scales of wellbeing: Johnson *et al.*, 2017). Similarly a range of qualitative research techniques have been employed to evaluate or understand the nature and meaning of art programmes, including interviews, focus groups, observations, reflective discussion, and individual journaling by artists and practitioners. Such practices have been bolstered by the development of robust frameworks and guidance for the design, collection and analysis of such qualitative data (e.g. Creative and Credible project: Daykin *et al.*, 2016; Dementia and Imagination project: Windle *et al.*, 2016). There are also a number of situations in which quantitative and qualitative methods are combined, including mixed-method approaches following the MRC framework for evaluating complex interventions ( Craig *et al.*, 2008), but in many scenarios qualitative input is limited to addressing the feasibility or acceptability of interventions.

All of our projects will seek to involve qualitative and quantitative evaluation following established guidelines ( Creswell, 2011). However we will also seek to develop new methodologies to address the complexities presented in capturing arts and dementia impact across different types of dementia at varying levels of severity.

One area of particular opportunity lies in developing tools to capture the dynamic quality of ‘in the moment’ responses. A number of observational scales record information about responses such as ‘positive affect’, but typically in the form of categorical data (present, absent) and at a single point in time (discrete rather than continuous measurement). Resolving such limitations would enable the enrichment of observational occurrence-type datasets by capturing important factors such as the duration, intensity, frequency and variability of such responses. Digital slider-based ratings of recorded video data by a range of observers may be one such approach (see Music for Life 360 description for further details). Dynamic, slider-based ratings may permit quantification not only of observable events (the traditional domain of quantitative research; e.g. ‘please rate the affect of the participant’) but also more qualitative phenomena which require interpretation on the part of the viewer/rater (e.g. ‘please rate how engaged you feel the participant was in the activity) or even the internalized generation of meaning within the viewer/rater (e.g. ‘please rate how connected you felt to the participant’).

## Overview of study themes

Created Out of Mind aims to demonstrate that the cultural and creative experiences of people with dementia can both challenge traditional definitions (=out of mind) and common perceptions/misconceptions (only affecting memory, the elderly), and stimulate curiosity about the healthy and aging brain.

The two primary themes of work are *People and Perceptions* and *In the Moment*.

Projects under the *People and Perceptions* theme aim to capture the narratives, questions, emotions and experiences of people affected by different forms of dementia, and to consider and respond to their representation across different media (traditional, social) and art forms (music, visual arts, etc). Through three-way conversations between people living with dementia, scientists and artists, the intention is to use scientific analysis and creative experimentation to empower people with dementia, open up new perspectives, and better inform representations of what it means to live with dementia. *People and Perceptions* projects are described in Box 1.

Box 1. **People and Perceptions project areas**

*Representations of dementias:* In collaboration with the Wellcome Library, this project will explore how ideas, attitudes to, and perceptions of dementias have been documented from the 19th century through to the present day. Different perspectives and judgements of dementias will be contracted from a variety of historical accounts and sources, encompassing both traditional medical texts and lived, personal experiences.

*Dementias in the media:* This project will use Natural Language Processing (NLP) to identify and analyse the language used by a variety of media platforms to discuss dementias (e.g. BBC, Daily Mail, Guardian, Sun, Twitter, Reddit). These analyses will both shed light on how the use of language affects prevailing attitudes to the dementias, and also guide the language to be used when communicating about dementia.

*Metaphors for the mind:* Building on previous social gerontological work considering dementia as a cultural metaphor ( Zeilig, 2014), this project will collect and analyse the visual, textual & linguistic metaphors used to describe dementia and provide an opportunity for people affected by dementias to respond to these descriptions. By empowering and integrating the multiple and unique voices of people living with dementias into public and scientific descriptions, the work will inform more inclusive representations.

*The art of conversation:* The verbal and non-verbal communication between care professionals and people with dementia, in particular people with advanced dementia, will be explored qualitatively through interviews with care staff, observation and natural language processing models. In-depth analysis will be conducted examining the role of an arts-based staff development intervention to improve communication and the experiences of people with dementias and the people that care for them.

*The whole is greater than the sum of its parts:* A review and meta-analysis of the main findings from each of the People and Perceptions projects will explore the challenges, differences and similarities between these elements at the end of the residency. It will explore how and where these projects can inform and influence perceptions and understanding of dementias and how they might be used in future to shape future communications; ultimately enriching the experiences of those living with dementias.

*In the Moment* projects are directed towards the development of methods and toolkits to evaluate the experience and impact of dementia-focussed arts activities. The projects will explore how people, with and without dementia, respond to different experiences such as seeing art and exhibitions, handling objects or hearing music. A central aim is to understand the benefits of combining established and new qualitative (descriptive) and quantitative (numerically based) techniques in a variety of settings. *In the Moment* projects are described in Box 2.

Box 2. **In the Moment project areas**

*Measuring the moment:* Measuring the response to participatory arts activities is complex, and many assessment tools and methods currently used to evaluate art programmes require further development and validation. This project will critically review current approaches to capturing the impact of, and responses to arts activities, and compare these approaches with those used by Created Out of Mind. This will enable us to assess the relative value of each approach.

*Measuring the response:* Arts interventions and interaction with the arts can create meaningful, positive experiences for people living with dementia, as well as improve quality of life. Qualitative techniques such as self-report effectively describe the emotional responses the arts can produce, for example when listening to or performing music. Physiological measurements such as stress hormone levels and galvanic skin response (a change in the electrical resistance of the skin caused by arousal) can be used to try and quantify such responses. When taken together, these can give a picture of the kinds of physiological outcomes that are associated with positive affect and improvements in mental wellbeing in the context of arts interventions. This review will aim to give a comprehensive overview of the studies which measure some form of physiological outcome in people living with dementia in response to the arts, or to an arts intervention. The review will indicate how further research in this area can help to broaden our understanding of the effects of the arts in the setting of dementia.

*Music for life 360:* Building on the Music for Life programme of improvisatory, participatory music ( https://wigmore-hall.org.uk/learning/music-for-life ), this project will use 360° video recordings to capture the musical and non-musical contributions, responses and interactions of participants and practitioners within a session. Groups of observers (including carers known to the participants and neutral observers) will subsequently rate factors such as engagement and emotion afforded by the video recordings of individuals or of groups of participants, using a novel slider-based continuous rating scale. Video analysis will also be used to identify relationships between musician and participant behaviours, one of which being the motivation to be involved in the session.

*Mind, body and song:* In this study the impact of choral singing on people living with and without a dementia will be evaluated by measuring physiological responses (e.g. stress hormones) and psychological responses (subjective anxiety, loneliness and subjective wellbeing) before and after a choral singing session. These before and after measures will be complemented by continuous measurement of other physiological variables (galvanic skin response, heart rate) during sessions. Identical measurements will also be made before, during and after participation in a control non-singing cultural participatory activity. Analysis will examine predictions that choral singing elicits a reduction in stress hormone levels and therefore a relaxation response, and that this is associated with decrease subjective anxiety and loneliness.

*Dementia-eye view:* Cortical visual impairment in dementia is an under-recognised factor influencing interactions with the physical environment. This study will examine how people with dementia-related visual impairment, caused by Posterior Cortical Atrophy (PCA) and typical Alzheimer’s disease (tAD), and their carers, navigate real-world environments, such as the Medicine Man gallery at Wellcome Collection. Subjective experience of artefacts and spaces within the gallery will be evaluated by audio recordings, and by filming from body-worn cameras and the use of a Think Aloud verbalising technique ( Gilhooly *et al.*, 2007). Physical position, location and movement will also be monitored continuously using ARCCS sensors (Accessible Routes from Crowdsourced Cloud Services), and physiological responses (heart rate, galvanic skin response) using Empatica E4 devices ( https://www.empatica.com).

*Play it again:* Music evokes many emotions and listening to music stimulates a physiological response. This study will capture multimodal physiological data in an effort to determine the most sensitive and relevant metrics for evaluating the impact of recorded music and detecting musical preference in individuals living with dementia. Participants will listen to excerpts of familiar and unfamiliar music (determined by personal selection, music one would expect people to be familiar with based on their life experiences,and experimental manipulation [e.g. spectral inversion]). Physiological metrics will include pupillometry, eye movements, heart rate, and skin conductance. Video recordings of facial expressions will also be subjected to facial emotion classifier algorithms. Machine learning techniques will be applied to determine patterns of response associated with familiar and unfamiliar music perception.

*Thinking eyes:* Through visual art, this project aims to understand the relationship between perception, identity and communication in people with different dementias. Individual and group sessions will be conducted in which participants with and without dementias are presented with various forms of visual art and complex imagery. Experimental conditions will aim to establish the impact of participants saying what they experience when viewing the images, hearing what others think, and hearing ‘expert’ factual commentaries. Responses to these conditions will be captured using qualitative and computational analysis of spoken output, eyetracking analyses of where people look and in what order, and physiological reactions to the experiences (pupillometry, heart rate, skin conductance). The broader aim is evaluate the impact of techniques designed to help people with dementias express their personal experiences and insights and to encourage people to rethink how they perceive art and the world.

*Colour Rooms:* Research with healthy people has shown that people prefer different colours in different contexts, but little is known about how variations in spatial and material properties might influence how people with dementias experience colour. The Colour Rooms project will explore these questions. In today’s digital world, the majority of contemporary research on colour is done using computers, but they lack the surface and texture of the real world. The first phase of the study will address this by presenting the Colour Rooms both as digital images on a computer monitor and as high quality photographic prints to study if people with dementia respond differently to them. In a later phase, participants will view larger scale constructions of colour in space, further exploring the differences between virtual and material experiences from a perspective of embodied cognition.

*Single yellow lines:* This project examines the effect different dementias have on the way people express themselves through gestures. Single Yellow Lines will involve participants painting two separate lines on two separate canvasses, one joining two dots by painting the straightest line possible, the second being the expressive decision of the participant. The lines can be painted at any speed but the execution should be a single action (or gesture) like a move in chess. Heart rate, galvanic skin response and physical limb and body position data will be gathered during the gestures, in addition to qualitative analysis of spoken descriptions of the participants’ experiences. *Single yellow lines* attempts to marry an experiential and experimental examination of clearly definable ‘moments’ in the context of the visual arts, with each individual mark a unique artistic reflection of the experience of that person at that time.

*Quality of life DIY:* Quality of Life (QoL) is a commonly considered outcome when evaluating arts and health programmes, but it is unclear how well standardised QoL measures capture the experiences of people living with rare and young onset dementias. This project will use a Q-sort methodology to enable participants to choose which scale items are most, least and somewhat important to them. The data gather will address questions such as: does memory matter more than mood? Are chores more important that hobbies? The project will consider ways to tailor other questionnaire measures for people with different dementias and in doing so to capture more of the quality of experience in our quantified scales.

*Things in our lives:* Cultural spaces such as museums and art galleries often have a variety of objects which they invite their visitors to explore. This project aims to explore the effect that viewing visual art and handling museum, and other, objects has on people living with dementias (e.g. related to wellbeing, language and memory), as well as what insights this can provide about people’s relationship with objects and cultural spaces.

The *People and Perceptions* and *In the Moment* themes are *inspired by* people living with dementia but do not have to be limited to being *about* dementia. Shaping public and professional perceptions of the dementias, and creating useful toolkits for evaluating dementia and arts practice are key goals, but not the only goals. *Created Out of Mind* also aims to generate work which questions our expectations of health, disease and creativity, and how we experience ourselves ‘in the moment’.

### ‘Methodology gap’ projects

A small number of additional projects are oriented more to engagement in and reflection on the research process, rather than directly addressing research questions. These projects are aligned with the aim of continuing to search for new ideas and ways of working throughout the duration of the residency, not just delivering defined projects conceived at the outset. We regard these projects (and elements of the collaborative development of other listed projects) as sitting within Arends’ and Thackara’s ‘methodology gap’.

In our previous practice, these moments of collaborative collision have most frequently occurred in the context of conversations between people living with dementia and clinician scientists. Examples include the puzzling uncertainties of individuals who described perceiving a room as entirely upside down or who were forced to enquire ‘Am I the right way up?’, which triggered a first exploration of balance deficits in a syndrome (posterior cortical atrophy; PCA) previously considered to be visual in nature (e.g. Crutch *et al.*, 2011). Descriptions of heightened or emerging artistic interest and competence in individuals with frontotemporal lobar degeneration (FTLD) have also led to explorations of emotional awareness using abstract artistic stimuli (e.g. Cohen *et al.*, 2016).

Examples of Created Out of Mind projects which intend to bring artists, scientists and people with dementia into these collaborative collisions are described in Box 3.

Box 3. **Methodology gap project areas**

*Talking Life:* There is a common assumption that people experiencing dementia become rapidly unable to engage in meaningful conversation. Talking life is a series of hosted podcast conversations with a person, or people, living with a dementia about their relationships with topics such as: desire, beauty, purpose, connectedness, and sleep. The idea is for listeners to engage in collective conversations with people experiencing dementias about everyday subjects, challenging them to reconsider their initial assumptions about dementias.

*Brains in a Dish:* Making use of induced pluripotent stem cell (iPSC) technology, artist, clinician and broadcaster members of the team will observe the transformation of their own skin cells first into stem cells and then into brain cells (neurons). The team members will then reflect on these processes with individuals living with inherited dementias who have been through similar processes for research into the neurodegenerative disease which affect their families. The project will inspire personal, scientific and creative observation and reflection.

*Co-Creativity:* The term ‘co-creativity’ has fairly recently come into use to describe a particular approach to participatory arts practice. Despite the sense that co-creativity is of increasing relevance to both artists and cultural institutions alike, it seems that the term has not been defined in terms of artistic practice with people living with dementia. This project seeks to explore the intricacies, challenges, possibilities and potential of working co-creatively with people with a dementia. As a first stage, team members are engaging in a reflective correspondence on the theme of co-creativity. This correspondence will form the backdrop to the shaping of an innovative co-creative project at the Hub with an interdisciplinary team of artists and a group of people living with dementia. People living with dementia will be integral members of this project. The project will add depth to our exploration of co-creativity and help to illuminate the logistics of co-creative work as well as the meanings associated with it both for artists and for participants.

*Testing situations:* Created Out of Mind is situated in a unique setting that allows access to various opportunities, disciplines, resources and expertise across UCL, Wellcome Collection and multiple academic, cultural and charitable institutions. Through these collaborations, the team plan to create a series of artworks, archives, installations and events taking inspiration from the historical and the present context of cognitive tests that measure our memory, language, thinking and perceptual skills. Another aspect is the critical analysis (both positive & negative) of testing as a site for diagnosis & as a measure of human worth (as well as health) - what are the implications when someone’s functional value has been challenged and undermined? Also the related/contrasting artworks and environments that we’ve uncovered open up exciting spaces for further work / collaboration.

## Researching the process, not just the projects

The process of conducting collaborative research in the Hub is as much a valued output as what might be construed as an academic, public or produced output emerging from the project’s central research questions. The importance of interrogating the collaborative process of the residency calls into question what an ‘output’ might in fact look like. The interrelations and politics of how a project like this works is therefore justifiably a field of research in its own right ( Callard & Fitzgerald, 2015). What does it mean to genuinely collaborate and what role does the social proximity of sharing a space have on lines of artistic and academic enquiry? What role does the space of the Hub play on this dialogue and how does sharing a space within a public building influence, challenge and play with the direction of research? This is important to situate within Created Out of Mind not just between science and art, but with the critical arts and humanities that straddle these ‘specialisms’.

These questions are at the heart of the Hub. Wellcome recently convened a wider network of other practitioners and conveners of collaborative, experimental and publicly situated work, whereby methodology was as much a subject of interest as the core questions of the project itself. As was shown, one of the most difficult things about collaborative working at scale is understanding and mapping how the inter-connection of individual relationships and their subsequent subtle but substantial impact may have on the research methodologies and outputs.

**Figure 2.**
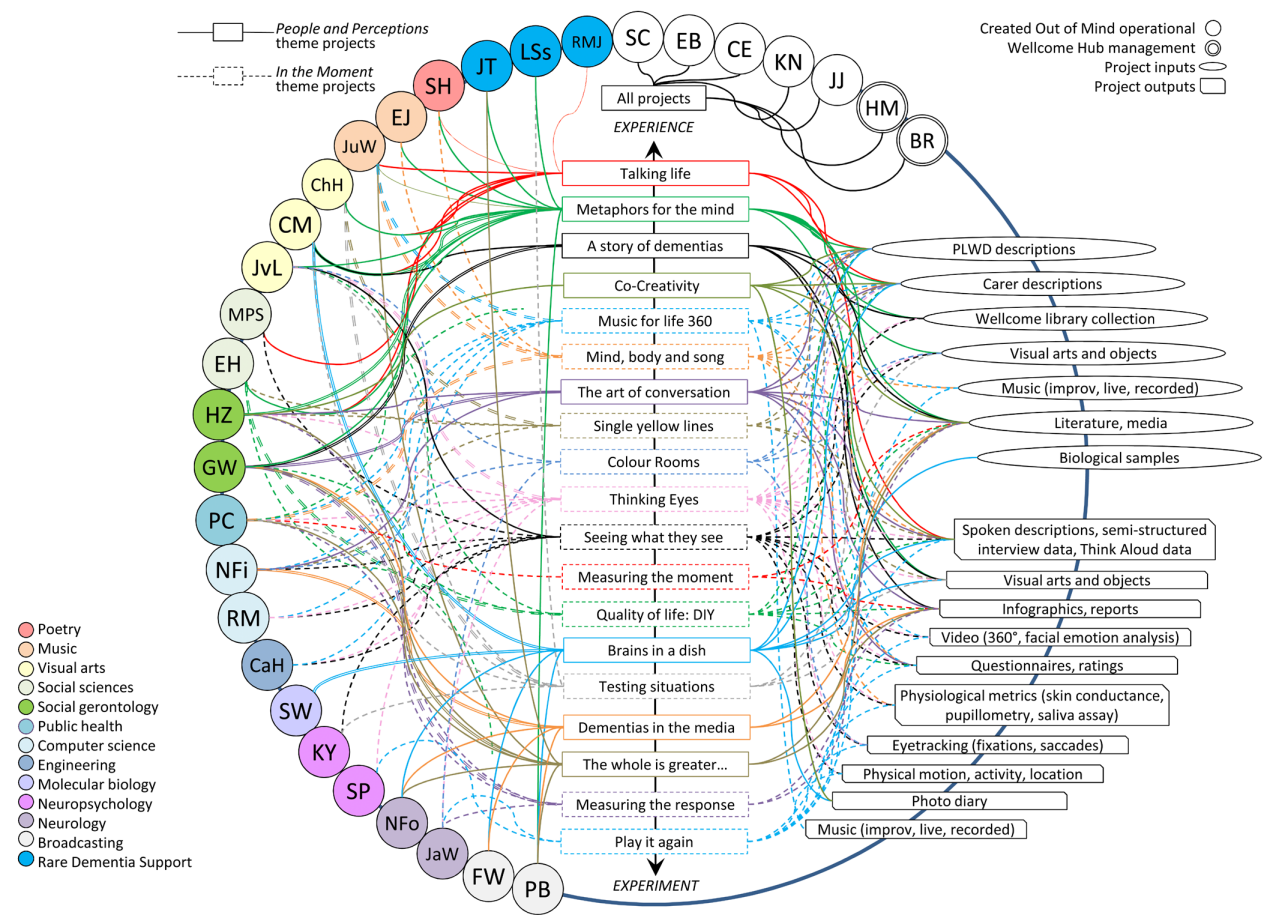
Illustration indicating the rich network of connections between collaborators and project content within Created Out of Mind. The connections of the teams working on each project are cross-discipline (see Left); the content of, inputs to and outputs of the projects themselves are cross-modal (see Right).

One of the exciting opportunities that Created Out of Mind has as the second residents of the Hub is to explore methods of interdisciplinary research and trial new ways of capturing and documenting this information. Residents past and present are encouraged to devise a number of projects to explore the mundane daily realities of collaborating working (e.g. a Diary Room ( Callard *et al.*, 2016) which randomly invited collaborators to reflect on their personal feelings towards the project; a re-construction of a medieval scriptorium whereby the team can scribe their reflections as the project continues). Created Out of Mind have already captured a baseline mapping of the group’s relationships during a gathering of the wider collaboration network immediately prior to taking up residency. Periodically mapping these connections in a visual matrix will show an evolving lattice of interconnectedness ( Figure 2). The Hub also houses an interactive visual timeline of the project, tracking these shifting perceptions, with the potential to indicate where and how the collaborative process has both deviated and expanded upon the project’s central inquiry. These observations formed important outputs of the project, as much as journal articles, performances and exhibitions, ones where the process of collaboration takes on a role of equal value to the more classically defined project outputs. The focus on these methods alongside the central research projects (see Box 1 and Box 2) allows Created Out of Mind to set their work in a distinct context within the wider research field of interdisciplinary methodology. Poised to champion and raise awareness of the crucial role that collaborative techniques play within their project, both the residents and the Hub space have a unique role to play in making visible these underlying mechanics that form the bones of experimental collaborative work.

## Study management

A study setup such as Created Out of Mind demands a nuanced approach when it comes to selecting the team involved in the project on a day-to-day basis. A truly interdisciplinary team requires a combination of individuals who are leaders in their respective disciplines; some of which more typically align with dementia research (e.g. neuropsychology) and others for whom this project provides an unusual opportunity to apply their areas of expertise to a relatively novel field. Inevitably, the focus on dementia in Created Out of Mind holds a strong personal connection for a number of our collaborators. Dementia is a hugely emotive and personal subject, and it is for this reason that we concluded in the early application stage that the study team would not be representative unless it included individuals who have received a diagnosis of dementia, carers and their closest relatives. These individuals contribute to and help shape our research questions and directions of enquiry with their feedback and involvement in the evolution of our projects (e.g. UCL Rare Dementia Support Group members).

The variety of disciplines and expertise coming together in Created Out of Mind highlighted the differences in the ways different individuals and institutions expect to be contracted to a large project, the agreement of which offers (to a number of individuals) a sub-award from UCL, the grantholding body, and governance concerning the generation of intellectual property (IP) and acknowledgements. The wish for us to expand, amend and emphasise was placed on different aspects of the agreement, largely by virtue of type of establishment. This propelled the contracting exercise from being simply administrative to one which provided fascinating insight into the differing priorities and pressures our differing collaborating establishments face within the contexts in which they operate.

The innovative nature of this project has and continues to generate a number of queries around governance and around conducting research with NHS patients in a cultural institution. The biggest governance challenge to date centres on obtaining approval from the Queen Square NHS Research Ethics Committee. By nature of this project resulting in a number of quantitative, qualitative and creative outputs, and in keeping with our goal of examining the arts and sciences with equal scrutiny, we took the decision to regard all outputs (traditional research outputs and creative outputs) as data, designing the application with this model in mind. As a proportion of the Residency centres on exploration, we submitted an upfront application which explained the unusual proposal and timeframe set by Wellcome for this particular study, acknowledging any ambiguous areas with a request to inform the Committee once confirmed. The protocol included only behavioural procedures, with arguably low-risk activities (e.g. passively recording heart rate while participants observed visual artistic images).

The NHS Research Ethics Committee expressed an unfavourable view primarily based on the extent to which the information we planned to collect could be regarded as research. It was an interesting reflection on both the way we approached our project specifically, and on the freedom of dementia research in general. This decision surprised us; particularly in light of funding bodies actively encouraging interdisciplinary investigations in order to pursue novel avenues for research. Della Sala & Cubelli (2016) note in their opinion piece ‘Entangled in an ethical maze’ the difficulty of certain models of studies to be considered, citing psychology and neuropsychology studies as including particularly difficult models or protocols for Research Ethics Committees to approve. A resubmission to the NHS Research Ethics Committee aligning the research project to the traditional structure has since gained ethical approval (17/LO/0099).

We are interested in how Created Out of Mind, a project free in many ways to explore dementia through the sciences and creative arts, is able to navigate the necessary parameters of research governance protocols so that both the project and the Research Ethics Committee can enjoy a compromise of balancing perceived patient safety and risk with research innovation. We welcome the opportunity to use the model of Created Out of Mind as a tool to encourage and progress the development of collaboration between Ethics Committees and researchers, artists, and practitioners investigating dementia ( Cubelli & Della Sala, 2015).

## Public engagement and dissemination plans

Created Out of Mind is uniquely positioned as both a research and public engagement residency; working in both capacities within the public museum building of our funder. Running in parallel to Created Out of Mind’s research projects is our public story, and the way in which we tell this story will play a significant role in achieving our mission statement of shaping public perceptions of dementias. Therefore, we need to make the best use of engagement opportunities and develop a consistent and authoritative voice in communications surrounding dementias. We have planned a series of events, press engagement, digital communications and campaigns to disseminate our research, and provide a platform for the voices of people living with dementias that work with us, and inspire our work.

As an interdisciplinary team of scientists, artists, broadcasters and people living with dementias, we have a valuable opportunity to develop novel approaches to public engagement and curate events and experiences from multiple perspectives that, subsequently, invite people to view dementias in a different way to the prevailing narrative. We have a mutually beneficial relationship with Wellcome; drawing on their expertise, vibrant spaces and engaged audiences to maximise the impact of our public engagement, whilst also attracting new audiences to Wellcome Collection and placing lived experiences at the centre of how we communicate our work; aligning with one of Wellcome’s key priorities.

To date, we have led six events at Wellcome exploring themes such as language, gesture, expression, vision and representation within the context of dementia. Through these events we have started new conversations with the public about what it means to live with a dementia and invited visitors to take an active role in helping us to explore and shape our research questions. This is also being achieved through digital communications such as contributor blog posts, social media campaigns and image galleries. Most recently, in recognition of Dementia Awareness Week 2017 we invited people living with dementia, carers, family members and friends to share images of themselves engaging with the arts on social media for our Dementia Arts Photo Challenge. This was well received and had a significant impact on our social media reach; for example our Instagram following increased by 50%. We hope to continue this momentum and repeat this campaign several times during our Residency.

We hope that our work will culminate in a final event or exhibition that aims to provide powerful insights into the experiences of people living with dementias - offering unique perspectives on the personal and scientific dimensions of these conditions through a range of formats, including arts installations, live experiments, film and audio pieces.

We recognise the risk of working in ‘a bubble’ and that reaching diverse publics (including people living with dementias) across age, ethnicity, location, belief systems, cultures, genders and socio-economic backgrounds, should be a key consideration in our engagement strategy. This is a challenge, not just for us, but for many organisations working in this field. In order to tackle this, we have put together a separate ‘Public Engagement Fund’ to encourage researchers to take their work outside of Wellcome and across various locations in London and the UK. We have expanded our dialogue with dementia services in diverse and local London communities and met with people living with dementias to gauge feedback on our projects. This included meeting with the Alzheimer’s Society Service User Review Panels; two have been held so far in South London and Horsham in West Sussex and will be producing a written account of this work.

Our press engagement strategy also reflects our aim to reach as wide and diverse an audience as possible. We have journalist Fergus Walsh and science writer Philip Ball on the core team who help to ensure our research vision is effectively represented across press platforms. Our work has already been featured in widely-circulated publications including The Guardian (Ball, Philip, "Forgetting but not gone: dementia and the arts." The Guardian, 11th March 2017) and The World of Interiors (Van Leeuwen, Janneke, “Opening the Mind’s Eye.” The World of Interiors, August 2017). We aim to engage with a wide range of print and broadcast media to better inform how dementia is represented across different audiences and we will have a significant role in the upcoming BBC Radio 3 residency at Wellcome in October 2017. Through this residency, Wellcome and the BBC aim to introduce new audiences to their channels through wide-reaching advertising campaigns, and we hope to leverage on this to attract audiences not typically engaged with the topic of dementia.

There are also challenges that come with being a research team, working in public as opposed to a closed professional space. The idea of collective communications activity is a novel experience for many Created out of Mind collaborators. Approaches to, and ideas of what public engagement should achieve, vary across disciplines. Through this residency we will explore the opportunities, challenges and misunderstandings that may arise when approaching public engagement in this collaborative way and consider how we can use our collective voice to the best effect. We hope this will serve as a useful toolkit for future research teams of this nature.

## Summary and next steps

Created Out of Mind aims to deepen our understanding of how the arts are capable of engaging and enriching the lives of people living with dementia, and communicating about both the lived experience and biology of these conditions. A number of challenges lie ahead of the residency team – to ensure our work is authentic, rigorous, creative, and most of all of value to the dementia, arts and science communities. Although this residency focuses on research, rather than on production, it will be particularly important to ensure that our outputs are both artistic and scientific in nature. There will be tensions to be held, both in balancing the importance and demands of research discovery and the engagement of considered audiences, and in our ways of working with each other and with the space in which the work is situated. But the Hub residency offers a rich opportunity to engage in collaborative, experimental, publicly-situated research, which we hope will have a meaningful impact upon the dementia and arts field.
