# Supplementary material for: Preparatory planning framework for Created Out of Mind: Shaping perceptions of dementia through art and science
Source: Wellcome Open Res. 2017 Nov 6;2:108. [Version 1] doi: 10.12688/wellcomeopenres.12773.1 (PMC5710169; doi:10.12688/wellcomeopenres.12773.1)
Supplement: Peer review history

# Peer review: Referee response for version 1

**DOI:** 10.21956/wellcomeopenres.13838.r27628

**Published:** 2017-11-27

**Authors:** Jane Macnaughton

**Affiliations:** 1 Centre for Medical Humanities and Department of Anthropology, Durham University, Durham, UK

The Created out of Mind team in this paper take us on an exploration of how they have set out on their two year residency in the Hub space at Wellcome Collection. The residency sets out to ‘explore, challenge and shape perceptions and understanding of dementias through science and the creative arts’. They bring together scientists, clinicians, artists, and experts in communicating with the public in order to take this forward. 

 They are the second group to occupy the Hub and it seems from this account that they are building on the work of Felicity Callard and her team who were the first occupants of the space with the ‘Hubbub’ project. They draw upon Callard’s reflections on the essential messiness of the interdisciplinary process and also on questions of what ‘outputs’ from such research, carried out in a public space, might look like. The language used of the interdisciplinary process is striking: ‘troubling middle ground’, building on the ‘discomfort’ of individuals in this conflicted, collaborative space. Such language was apparent at the Hub workshop that is mentioned in the paper. Some attending that event even went so far as to speak about ‘fear’, and the ‘danger’ inherent to interdisciplinary working. I wonder how accurate this language is about what is actually going on in this space? I sense that researchers would not willingly submit themselves to being fearful or troubled. What seems more accurately to represent what it feels like to do genuine interdisciplinary work is the sense of excitement generated by the creative tension of being in dynamic conversation about a topic of common interest with others from widely different perspectives. This feels positive and thrilling – good reasons why people want to do it! The team describe spending the first three months of their residency developing their protocol, making use of the space and the opportunity for time to actually work together. Part of this process involved facilitation. My own experience in interdisciplinary work is that good facilitation throughout the process enables a positive, dynamic space to be created within which the team feel supported and, indeed, safe. 

 Interdisciplinary process is one key element of the residency, and I look forward to further reflections on how this evolves. Another element is engagement. The project essentially involves dynamic interaction between the arts, scientists and people with dementia. It was clear in the authors description of their ‘people and perceptions’ theme how they were to study dementia sufferers’ responses to art but it was less clear if and how they were exploring creative art’s response to dementia. This may emerge as the team start to develop their public engagement, but it would be interesting to see a genuine process of two-way engagement developing. 

 The residency protocol involves a second theme that I found very exciting: ‘in the moment’. This is an inspired idea, working with the notion that people with dementia often do live ‘in the moment’, and that should not be seen as a restriction but something to build on creatively. This reflects the aims of the residency to shift ideas about dementia signifying the end of life preceded by social death. Rather, in keeping with many chronic diseases, people live on with this problem and want to live well. I much look forward to seeing how this part of the research develops.

 As well as this crucial aim in relation to public consciousness, the Hub team recognise the potential of their project to take research into of the role of arts in health and wellbeing to an evidential level that will at last provide some basis for the efficacy that so many of us have observed in practice. Here they are asking some critical questions, especially ‘how long do benefits last’? Arts interventions in health care have so often been piecemeal and researched in a fashion that provides tentative evidence for short time (in the moment) benefits. It will be a real contribution if this team can show what role arts-based interventions can have on enabling those with long term conditions to live more fulfilling lives. 

 In view of the emergent nature of the Created out of Mind team’s research protocol - appropriately in keeping with the spirit of the Hub space - it was no surprise that they came up against a problem with NHS Ethics approval. I applaud their initial instinct to include ‘all outputs (traditional research outputs and creative outputs) as data’ and it is a pity that approach did not succeed. It is important that projects in medical humanities challenge structures that obstruct research taking creative new directions, particularly in conditions that have proved refractory to traditional treatment approaches. 

 This is an exciting new project that has the potential to enhance the lived experience of dementia, but also to move forward interdisciplinary, engaged research and I look forward to hearing more.

I have read this submission. I believe that I have an appropriate level of expertise to confirm that it is of an acceptable scientific standard.

# Peer review: Referee response for version 1

**DOI:** 10.21956/wellcomeopenres.13838.r27626

**Published:** 2017-11-21

**Authors:** Ken Arnold

**Affiliations:** 1 University of Copenhagen, Copenhagen, Denmark; 2 Wellcome Trust, London, UK

For the last three or four years, something of a socio-intellectual experiment has been conducted at the top of Wellcome Collection on London’s Euston Rd. Called the Hub, a space has been set up for a cohort of grant holders (awarded up to £1m over two years) to probe their chosen topic through a porous interaction between research, museum culture, and public engagement. Calling themselves ‘Hubub’, the first incumbents explored the concept of rest and its opposites, particularly as manifest in contemporary urban society. The second group reporting here (Created out of Mind) are interested in applying bifocal lenses of art and science to deepen our understanding of dementia, as well as to challenge commonly held perceptions about the condition. Around a third of their way down a carefully crafted path, this paper presents something of an interim posting about their journey thus far.

 An introductory section about the subject leaves us in no doubt of its critical importance. Few aspects of our aging societies are not having to grapple with the effects of this increasingly prevalent condition, made all the more challenging because of the shift in emphasis from simply ‘living longer’ to a more complicated desire of ‘living well’. Dementia then is one of our most pressing healthcare challenges, which can only be dealt with, Brotherhood *et al.* argue, through the development of new thinking and methodologies. At the centre of their project is a clear and innovative proposition: “creative arts are a powerful vehicle for understanding, communicating and navigating this dramatic cultural shift.”

 Created out of Mind sets out to integrate insights from science and the creative arts, and suggests that the new comprehensions anticipated are likely to result in shifts in therapeutic approaches, and the development of new tools for understanding as well as communicating about these forms of mental ill-health. These developments might well also have practical and economic impacts. Furthermore, working in the opposite direction, this ambitious project also holds out the promise of unlocking more general learning “about art, consciousness and the brain from the experiences [but also the brains] of people with dementia”.

 Novel ways of examining how people respond to art through the senses, and how they participate in creative arts and explore cultural spaces are being developed. To this end, they outline two broad thematic strands of enquiry. The list of sub-topics and proposed investigative methods gathered under each are ambitious, verging on daunting.

 The *People and Perceptions *strand aims to “capture the narratives, questions, emotions and experiences” of people with dementia and reflect on their representation in different media. Empirical material will be amassed by delving into the Wellcome library’s collections; through the application of Natural Language Processing techniques; by collecting relevant metaphors used in visual as well as textual forms; and by interviewing and observing the work of care-staff, focussing particularly on the language they use and the potential implications of using art in this relationship.

 Alongside that, the *In the Moment *strand seeks broadly to measure dementia patients’ responses to participating in the arts, initially by surveying and compiling findings from existing studies processed through meta-analysis. Additional, more focused, studies will include the examination of: the physiological effects of participating in choral singing; modes of navigating real-world cultural environments; colour preferences both in virtual and real environments; and the impact of museum material – i.e. objects. Much of this study aims deftly to combine experiential and experimental examinations, where possible, turning the public venue (Wellcome Collection) into a sort of real-life laboratory. A third layer to the project ( *Methodology gap*) involves rather fewer projects focused more on the interdisciplinary research process itself than directly on the core research questions.

 As the paper’s title makes clear the concern here is to describe and reflect on the projects preparatory planning frameworks. We will have to wait a while for the presentation of even initial findings, and that is tantalising. In the meantime, we learn how “individuals who have received a diagnosis of dementia, carers and their closest relatives” are involved in the project as far more than just subject participants. Questions about how to balance classically ‘objective’ scientific studies with other more ‘subjective experiments’ involving patients investigated in closer-to-real environments are touched on. And so too are broader philosophical questions about consciousness (our experience of ourselves ‘in the moment’) and our shifting expectations of health, disease and creativity. Though necessarily restricted to the preparatory and aspirational, the material is nevertheless presented in a clear, honest and thoughtful manner.

 One particularly well-made methodological point distinguishes the need for a ‘searching’ phase prior to any research. This is a crucial but distinctly under-investigated or articulated process during which hypotheses emerge. Where actually do those hunches, itches, and puzzles come from and how are they developed? And can a project where art (typically imagined as an organically evolving exploration) intermingles with science (traditionally described in terms of executing a pre-determined set of aims, procedures and analyses) across a ‘methodology gap’ provide some insight here? This is decidedly a paper that asks questions rather than answering them; but it is striking here that the concept of curation seems particularly useful.

 Most of all, this is a paper that gets us to think in close proximity about how a substantial piece of self-consciously interdisciplinary work is actually undertaken. It includes two diagrams, which even when expanded to full screen, still present a dizzying array of intricate inter-connections between sub-themes and discrete packages of work. Those who have been involved in this type of project (one that has gained significant traction in the last decade or two) will recognise in them the almost overwhelming complexity of what is being attempted: laboratory habits applied to ex-laboratory conditions, efforts to make qualitative and quantitative evidence square up to each other; an eagerness for human subjects and experiences not to be taken for granted or explained away; and an urgent need to be as interested in processes as in goal-driven products.

 As well as a spirited and impressive statement of intent, we are also given insight into how initial challenges have been dealt with. So underlying tensions are hinted at, ones which highlight ways that “different individuals and institutions expect to be contracted to a large project”, as well as a political dimension to how disciplines relate to each other, both of which can result in new creative insights. Even more tellingly, the difficult process of obtaining approval from the Queen Square NHS Research Ethics Committee is outlined – their initial rejection coming as a ‘surprise’. Research governance protocols are, however, established to maintain standards and minimise risks, so maybe this initial response is itself a measure of the innovations being attempted here. They also elicit from Brotherhood *et al.* the productive suggestion of employing a more collaborative model for making submissions to such a body.

 But ultimately, like the anticipated positive effects of using art with dementia patients under investigation, the new ways of thinking and working that this group are employing have not quite yet landed. They know the importance of genuinely collaborative practices and the potential significance that pursuing them in a shared space that allows social proximity between lines of artistic and academic enquiry as well as lived experiences of dementia; but they aren’t yet able to demonstrate that. We can anticipate proof of new insights about the role of art in dealing with dementia and of the value of interdisciplinary research practices arriving together further down the line.

I have read this submission. I believe that I have an appropriate level of expertise to confirm that it is of an acceptable scientific standard.
